# Allergies to antibiotics among US women with uncomplicated urinary tract infection

**DOI:** 10.1371/journal.pone.0304318

**Published:** 2024-09-26

**Authors:** Ashish V. Joshi, Alen Marijam, Fanny S. Mitrani-Gold, Jonathon Wright

**Affiliations:** 1 GSK, Collegeville, Pennsylvania, United States of America; 2 GSK, Wavre, Belgium; 3 Cerner Enviza, Malvern, Pennsylvania, United States of America; University of Cape Coast College of Health and Allied Sciences, GHANA

## Abstract

Uncomplicated urinary tract infections (uUTI) are generally treated empirically with antibiotics. However, antibiotic allergies limit the available oral treatment options for some patients. We assessed the proportion of self-reported antibiotic allergies among US women with uUTI. We performed a cross-sectional survey of US women (≥18 years) with a self-reported uUTI in the previous 60 days and an oral antibiotic prescription. Participants completed an online questionnaire about their most recent uUTI episode. Descriptive self-reported allergy data were stratified into subgroups by whether the participant had recurrent UTI (≥2 uUTIs in the past 6 months or ≥3 uUTIs in past 12 months, including the index episode), the number of different antibiotics given for the index episode (1, 2, ≥3), and whether the treatment was clinically aligned according to Infectious Diseases Society of America uUTI guidelines. Overall, 375 participants completed the questionnaire. The most commonly prescribed antibiotics were trimethoprim-sulfamethoxazole (SXT; 38.7%), ciprofloxacin (22.7%), and nitrofurantoin (18.9%). Most participants (62.7%) received only 1 antibiotic for their uUTI, and most (56.5%) were classified as having a non-recurrent uUTI. No antibiotic allergies were reported for most participants (69.3%), with 24.0% reporting 1 antibiotic allergy and 6.7% reporting ≥2 antibiotic allergies. Allergies to ≥2 antibiotic types were more common among participants classified as having recurrent uUTI, or who used multiple antibiotics to treat their uUTI. The most common allergy was to SXT (15.7%), followed by amoxicillin-clavulanate (8.3%) and ciprofloxacin (5.3%). Similar allergy trends were seen across subgroups, except higher rates of ciprofloxacin allergy were seen in participants given multiple antibiotics. Antibiotic allergies were relatively frequent in this uUTI cohort and the most common allergy was to SXT, which was the most prescribed antibiotic. Allergies to antibiotics reduce the available treatment options for uUTI in some patients.

## Introduction

Uncomplicated urinary tract infections (uUTIs) are one of the most common community-acquired infections in women [1]. Treatment for uUTI is typically empiric with the prescription of oral antibiotics. Thus, a substantial proportion of antibiotics prescribed in the outpatient setting are for the treatment of uUTI [2]. Antibiotics are the most common cause of drug-related hypersensitivity reactions [3], often leading to treatment discontinuation and the need for at least one course of an alternative antibiotic, which may both be complicated by, and contribute to, antibiotic resistance [4]. Documented antibiotic allergy labels in patients’ health records limit available treatment options for some patients and result in treatment changes such as the displacement of first-line agents and use of broad-spectrum antibiotics [3]. We examined the prevalence of self-reported antibiotic allergies among women with uUTI in the United States (US).

## Materials and methods

This was a cross-sectional online survey conducted to better understand the key drivers of health-related quality of life, work productivity loss, healthcare resources use, costs, activity impairment, and treatment satisfaction of women with uncomplicated UTIs; primary results from this study have been published previously [5]. Eligible participants were English-speaking, female US residents aged ≥12 years (though no responders were <18 years of age) who self-reported suffering a uUTI in the 60 days prior to their involvement in the survey, and who received an oral antibiotic treatment for uUTI. The surveys were conducted in the US between July 28 and September 28, 2020.

Participants were sourced from among individuals in the US who had previously agreed to take part in general population surveys fielded by the following panel companies: Dynata, EMI, Lucid/Federated, and Kantar Profiles. Participants were originally recruited to these survey populations through a variety of methods, including traditional and online advertising, affiliate networks, direct emailing, and word of mouth. Demographics for each company’s survey populations compared with the 2019 US Census have been published previously [5]. All participants provided informed consent online prior to their involvement in the study. Participants received compensation for the time required to complete the survey.

To ensure that participants were reporting uUTI rather than complicated urinary tract infection (UTI), participants were excluded if they reported any of the following in the 6 months prior to index treatment: urologic or ureteral abnormalities, interstitial cystitis, pyelonephritis, kidney stones, or renal failure. Participants were also excluded if they were asymptomatic when diagnosed with a UTI (i.e., only diagnosed due to a positive urine culture with no other UTI symptoms present) and if they reported: uncontrolled diabetes, immunosuppressant use, UTI/treatment occurring during inpatient hospitalization, pregnancy during UTI/treatment, organ transplant, or neurological disease.

Participants were invited to complete the survey via email. An online screener was built into the web-based survey such that all patients were screened using the same inclusion and exclusion criteria. After screening, participants completed the survey online with reference to their most recent uUTI (index uUTI). Broadly, the questionnaire covered participant demographics, health characteristics, uUTI history, uUTI symptoms, activity and work impairment, uUTI treatments and treatment satisfaction, antibiotic use, health status, and healthcare resource use and costs; the full questionnaire has been included in the Supplement. The current analysis focused on participants’ responses regarding history of uUTI recurrence (≥2 UTIs in the past 6 months or ≥3 UTIs in the past 12 months, including the index episode), any history of known allergies to medications used to treat uUTI, and whether the last antibiotic received for a uUTI was a first- or second-line therapy. First-line uUTI antibiotics probed by the questionnaire were trimethoprim-sulfamethoxazole (SXT), nitrofurantoin, and fosfomycin. Second-line antibiotics were ciprofloxacin, ofloxacin, levofloxacin, amoxicillin-clavulanate, cefdinir, cefaclor, cefpodoxime-proxetil, and cephalexin.

Self-reported history of allergy data were stratified by recurrent UTI (yes/no), number of different antibiotics given for index uUTI (1, 2, or ≥3), and whether the treatment regimen was aligned (1 first-line antibiotic) or not aligned (1 second-line antibiotic or multiple antibiotics [any line]) with Infectious Diseases Society of America uUTI treatment guidelines [6]. Recurrent UTI has previously been associated with lower patient quality of life [5], and the need for multiple antibiotics and non-adherence to guidelines may suggest ineffective initial therapy. Data are reported descriptively as frequencies and percentages, no formal statistical tests were performed.

Ethical approval for the study protocol was provided by Pearl IRB LLC (Indianapolis, US; reference #20-KANT-222). Participants provided written informed consent (via an online consent form) prior to their involvement in the study. No personally identifiable information was collected as part of the study. The study complied with all applicable privacy laws.

## Results

Overall, 54,020 individuals accessed the survey questionnaire, 387 passed the screening questions and completed the survey; 12 participants were excluded due to invalid or missing data. A total of 375 participants were eligible and completed the survey. The high rate of screening failure relates to the conservative approach taken to ensure that participants included had uUTI as opposed to asymptomatic bacteriuria or complicated UTI. Participants were mostly White (84.8%), from the Midwest (44.3%), Northeast (20.0%), South (20.0%), and Western (15.7%) US. Most participants received only 1 antibiotic for their index uUTI (62.7%) and just over half were classified as having non-recurrent uUTI (56.5%). The most commonly prescribed antibiotics for the index uUTI were SXT (145/375, 38.7%), ciprofloxacin (85/375, 22.7%), nitrofurantoin (71/375, 18.9%), cephalexin (56/375, 14.9%), and amoxicillin-clavulanate (35/375, 9.3%).

**Fig 1:**
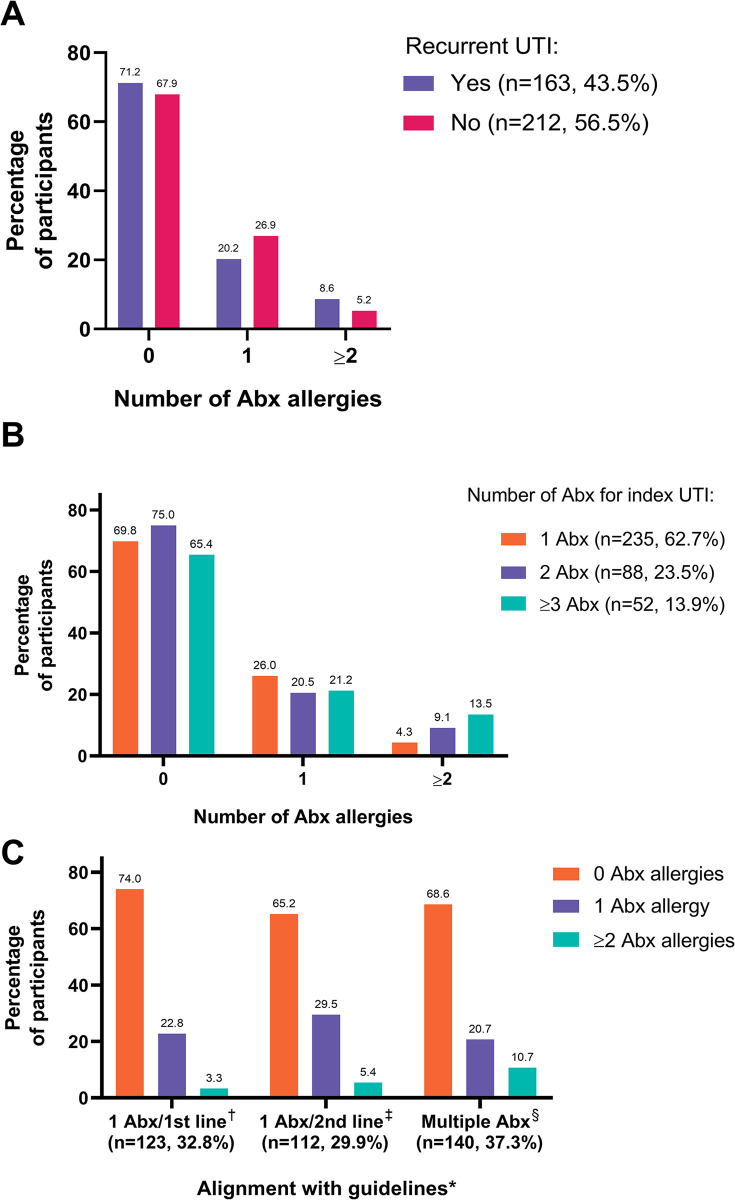
Proportion of participants with antibiotic allergies across stratification subgroups: Recurrent uUTI (A), Number of antibiotics received (B), and Appropriateness of therapy (C). *Alignment with guidelines: 1 first-line antibiotic; not-aligned treatment: 1 second-line antibiotic or multiple antibiotics (any line). Defined according to guidelines for uUTI from the Infectious Diseases Society of America^5^; ^†^Defined as only 1 first-line oral Abx used to treat the index uUTI; ^‡^Defined as only 1 second-line oral Abx used to treat the index uUTI; ^§^Defined as 2 or more different oral Abx (any line) used to treat the index uUTI. Abbreviations: Abx, antibiotic; UTI, urinary tract infection; uUTI, uncomplicated urinary tract infection.

Most participants reported no antibiotic allergies (69.3%), while nearly one-quarter of the cohort (24.0%) reported one allergy, and 6.7% reported ≥2 allergies. When stratified by uUTI recurrence, 8.6% of participants with recurrent uUTI reported ≥2 allergies while the rate was 5.2% in those with non-recurrent uUTI. A lower proportion of participants with recurrent uUTI reported 1 allergy (20.2%) than those with non-recurrent uUTI (26.9%) (Fig 1A). The proportion of participants reporting ≥2 antibiotic allergies was higher with an increasing number of antibiotics prescribed for the index uUTI (1: 4.3%; 2: 9.1%; ≥3: 13.5%) (Fig 1B) and with treatment not aligned with guidelines at index, most notably in the multiple antibiotics category (10.7%) (Fig 1C).

The most frequently reported allergy was to SXT (15.7%), followed by amoxicillin-clavulanate (8.3%) and ciprofloxacin (5.3%) (Table 1); among participants with recurrent uUTI, the rates were 17.2%, 9.8%, and 6.1%, respectively. The percentages of allergy to ciprofloxacin in participants who received multiple antibiotics for their index uUTI were: 5.7% and 11.5% of those receiving 2 and ≥3 antibiotics, respectively; and 3.8% for participants receiving just 1 antibiotic for their index uUTI. This was reflected by 7.9% of participants in the multiple antibiotics treatment category reporting ciprofloxacin allergy (vs. 4.9% for participants with guideline-aligned treatment) (Table 1); similar patterns in antibiotic allergy frequency were observed across the other subgroups.

**Table 1:** Frequency of allergies to specific antibiotics across stratification subgroups.

Abx allergies, n (%)		Recurrent uUTI		Number of Abx for recent uUTI			Regimen alignment with IDSA guidelines*		
		Yes (n = 163, 43.5%)	No (n = 212, 56.5%)	1 Abx (n = 235, 62.7%)	2 Abx (n = 88, 23.5%)	≥3 Abx (n = 52, 13.9%)	1 Abx/ 1st line^†^ (n = 123, 32.8%)	1 Abx/ 2nd line^‡^ (n = 112, 29.9%)	Multiple Abx^§^ (n = 140, 37.3%)
**Any allergy**	115 (30.7)	47 (28.8)	68 (32.1)	71 (30.2)	26 (29.5)	18 (34.6)	32 (26.0)	39 (34.8)	44 (31.4)
SXT	59 (15.7)	28 (17.2)	31 (14.6)	33 (14.0)	18 (20.5)	8 (15.4)	14 (11.4)	19 (17.0)	26 (18.6)
Amoxicillin-clavulanate	31 (8.3)	16 (9.8)	15 (7.1)	21 (8.9)	4 (4.5)	6 (11.5)	6 (4.9)	15 (13.4)	10 (7.1)
Ciprofloxacin	20 (5.3)	10 (6.1)	10 (4.7)	9 (3.8)	5 (5.7)	6 (11.5)	6 (4.9)	3 (2.7)	11 (7.9)
Cephalexin	14 (3.7)	6 (3.7)	8 (3.8)	9 (3.8)	2 (2.3)	3 (5.8)	5 (4.1)	4 (3.6)	5 (3.6)
Nitrofurantoin	7 (1.9)	1 (0.6)	6 (2.8)	6 (2.6)	0	1 (1.9)	2 (1.6)	4 (3.6)	1 (0.7)
Levofloxacin	5 (1.3)	3 (1.8)	2 (0.9)	2 (0.9)	1 (1.1)	2 (3.8)	0	2 (1.8)	3 (2.1)
Cefaclor	4 (1.1)	1 (0.6)	3 (1.4)	4 (1.7)	0	0	3 (2.4)	1 (0.9)	0
Ofloxacin	4 (1.1)	0	4 (1.9)	2 (0.9)	2 (2.3)	0	0	2 (1.8)	2 (1.4)
Cefdinir	3 (0.8)	3 (1.8)	0	1 (0.4)	2 (2.3)	0	0	1 (0.9)	2 (1.4)
Cefpodoxime-proxetil	2 (0.5)	0	2 (0.9)	1 (0.4)	1 (1.1)	0	0	1 (0.9)	1 (0.7)
Fosfomycin	0	0	0	0	0	0	0	0	0

Abbreviations: Abx, antibiotic; IDSA, Infectious Diseases Society of America; SXT, trimethoprim-sulfamethoxazole; uUTI, uncomplicated urinary tract infection.

*Appropriate treatment: 1 first-line antibiotic; inappropriate treatment: 1 second-line antibiotic or multiple antibiotics (any line). Defined according to guidelines for uUTI from IDSA^5^

^†^Defined as only 1 first-line oral Abx used to treat the index uUTI

^‡^Defined as only 1 second-line oral Abx used to treat the index uUTI

^§^Defined as 2 or more different oral Abx (any line) used to treat the index uUTI.

## Discussion

Allergies may limit the availability of oral antibiotics for empiric use in the treatment of common infections in some patients. The overall rate of known antibiotic allergies in our cohort of women with uUTI was relatively high, with nearly 1 in 3 (30.7%) reporting at least 1 allergy. The most common allergy was to SXT, which was also the most commonly received treatment. That the highest allergy rate was for SXT is in agreement with previously published data; however, our results suggest a much higher rate of allergy to this antibiotic: 15.7% compared with previous reports of 3–8% [7]. Higher rates of allergy to ≥2 antibiotics were generally found with UTI recurrence (vs. non-recurrent) and with receipt of multiple antibiotics (vs. just 1) to treat the most recent uUTI.

Previous research has typically used patient health records to determine antibiotic allergy rates; however, allergies to antibiotics are often not accurately recorded in health records [8,9] and thus rates determined in this manner likely under-report the true level of allergy for a given antibiotic. In our study, antibiotic allergies were self-reported by participants who may recall their own allergies more accurately than is recorded in their health record. The higher rates reported herein may therefore be more representative of the true rates of antibiotic allergies in women with uUTI.

We acknowledge, however, that patient-reported data has some inherent limitations, such as recall bias. Allergy information was self-reported without validation from a physician, and so participants may have mischaracterized adverse events experienced while receiving antibiotic treatment (drug-related or otherwise) as allergic reactions, as demonstrated previously for penicillin [10]. Additionally, there is a possibility of incomplete reporting of allergies as patients were asked to report known allergies to a pre-defined list of antibiotics used for uUTI. A further limitation of this study was that the survey was offered only to US-based English-speaking individuals previously registered with the survey companies. Therefore, the results may not be representative of all US patients with uUTI, including non-English-speaking patient groups, or groups who may be less willing or motivated to participate in online surveys, or whose health condition prevents them from doing so.

The high rates of antibiotic allergy found in our study underscore the problem of these drug reactions. Antibiotic allergy can be life-threatening and result in anaphylaxis and severe cutaneous reactions. Moreover, the consequences of antibiotic resistance may include increased use of alternative broad-spectrum and non-β-lactam antibiotics, which are associated with greater adverse events and antibiotic resistance [11]. Overall, our findings highlight the need for a greater range of oral therapies for uUTI to reduce the likelihood of treatment options being limited due to antibiotic allergy.
